# Acute myeloid leukemia in the next-generation sequencing era: Real-world data from an Austrian tertiary cancer care center

**DOI:** 10.1007/s00508-024-02463-w

**Published:** 2024-11-11

**Authors:** Sonja Wurm, Michael Waltersdorfer, Simone Loindl, Jennifer M. Moritz, Sereina A. Herzog, Gerhard Bachmaier, Andrea Berghold, Karl Kashofer, Christine Beham-Schmid, Gerald Hoefler, Hildegard T. Greinix, Albert Wölfler, Andreas Reinisch, Heinz Sill, Armin Zebisch

**Affiliations:** 1 Clinical Division of Hematology, Medical University of Graz, Auenbruggerplatz 38, 8036 Graz, Austria; 2 Institute for Medical Informatics, Statistics and Documentation, Medical University of Graz, Graz, Austria; 3 Diagnostic & Research Institute of Pathology, Medical University of Graz, Graz, Austria; 4 Department of Blood Group Serology and Transfusion Medicine, Medical University of Graz, Graz, Austria; 5 Division of Pharmacology, Otto Loewi Research Center for Vascular Biology, Immunology, and Inflammation, Medical University of Graz, Graz, Austria

**Keywords:** AML, Mutational profile, Controlled clinical trials, Real-world data, Leukemia biobank

## Abstract

**Background:**

Next-generation sequencing (NGS) has recently entered routine acute myeloid leukemia (AML) diagnostics. It is paramount for AML risk stratification and identification of molecular therapeutic targets. Most NGS feasibility and results data are derived from controlled clinical intervention trials (CCIT). We aimed to validate these data in a real-world setting.

**Patients, materials and methods:**

This study retrospectively analyzed 447 AML patients treated at an Austrian tertiary cancer care center. A total of 284 out of the 447 cases were treated between 2013–2023 when NGS was locally available for the clinical routine.

**Results:**

The NGS was successfully performed from bone marrow biopsies and aspirates, with processing times decreasing from 22 days in 2013/2014 to 10 days in 2022. Molecular therapeutic target(s) were identified by NGS in 107/284 (38%) cases and enabled risk stratification in 10 cases where conventional karyotyping failed. Concerning molecular landscape, *TET2 (*27%), *FLT3* (25%), *DNMT3A* (23%), and *NPM1* (23%) were most frequently mutated. Comparing older and younger patients (cut-off 70 years) showed enrichment in older people for mutations affecting DNA methylation (72% vs. 45%; *P* < 0.001) and the spliceosome (28% vs. 11%; *P* = 0.006) and more cellular signaling mutations in younger patients (61% vs. 46%; *P* = 0.022). Treatment outcomes corroborated a significant survival benefit in the recent NGS era and patients treated with novel/molecularly targeted drugs. Ultimately, biospecimens of these patients are stored within a leukemia biobank, generating a valuable tool for translational science.

**Conclusion:**

Our study validates data from CCIT and supports their relevance for treatment decisions in a real-world setting. Moreover, they demonstrate the feasibility and benefits of NGS within a routine clinical setting.

**Supplementary Information:**

The online version of this article (10.1007/s00508-024-02463-w) contains supplementary material, which is available to authorized users.

## Introduction

Acute myeloid leukemia (AML) is the most frequent form of acute leukemia in adults. While the prognosis is still dismal for many AML patients, the recent progress in diagnostics and risk stratification combined with the emergence and approval of novel therapeutic substances has significantly improved treatment outcomes for selected AML subgroups [1]. Most knowledge about AML diagnostic and treatment recommendations is based on controlled clinical intervention trials (CCIT). Despite all the advantages of CCIT, a downside of this approach is that specific patient groups are often excluded from these studies (i.e., frail, older and/or comorbid patients). Consequently, establishing real-world retrospective and prospective registries has evolved as an important quality control, yielding complementary value to the data from CCIT [2–5]. The relevance and transferability of CCIT to patient cohorts not represented in CCIT can be derived from these registries. Even for patient collectives included in CCIT, these registries enable an essential validation step within a real-world setting. Finally, they serve as essential quality assurance tools, enabling the comparison of treatment and outcome data between individual centers.

Next-generation sequencing (NGS) enables the parallel analysis of many genes in a high-throughput fashion [6, 7]. It revealed important insights into the pathogenesis of AML, which spurred the development of novel and targeted therapeutic approaches. Some of those, including the FLT3 inhibitors midostaurin [8] gilteritinib [9] and quizartinib [10], the IDH inhibitors enasidenib [11], olutasidenib [12] and ivosidenib [13] or the BCL2 inhibitor venetoclax [14], have already achieved approval by the U.S. Food and Drug Administration (FDA) and/or European Medicines Agency (EMA) as mono-treatment or combination treatment. Moreover, it refined AML diagnosis and risk stratification and is nowadays well implemented in the routine clinical work-up of AML. Even more, comprehensive NGS analysis is now a prerequisite to fulfilling the 2022 classification and risk stratification algorithms [1]. Most NGS data describing the molecular landscape of AML are derived from patient cohorts treated within CCIT. Given the recent establishment of NGS, real-world data about its feasibility in the clinical routine and data about the molecular landscape of patients not included in CCIT are scarce.

The Medical University of Graz, Austria (Med Uni Graz) is a tertiary cancer care center serving a population of approximately 1.2 million people. It was among the first hematology centers in Europe to include NGS profiling in the routine clinical diagnostic work-up of AML in 2013 [15]. Therefore, we aimed to perform a retrospective, unselected data analysis of AML in patients treated within the NGS era and to analyze epidemiological data and treatment outcomes as part of a real-world observational study. In addition, we analyzed the feasibility, usefulness and results of NGS, particularly in patient groups not represented in CCIT. Moreover, we used this registry to determine potential progress in AML treatment compared to data before the NGS era and/or before the introduction of recently licensed novel treatment approaches.

## Patients, material and methods

The study cohort consisted of unselected consecutive adult patients treated for AML between January 2013 and April 2023 at the Division of Hematology of the Med Uni Graz. Diagnostics and risk stratification of myeloid neoplasms were performed according to standard criteria [1]. To evaluate AML treatment progress over time, we also collected data from unselected consecutive adult AML patients treated at the same institution between 2002 and 2008. For NGS, genomic DNA extracted from bone marrow (BM) aspirates or biopsies was analyzed for mutations in up to 49 myeloid-associated genes using an Ion Torrent next-generation sequencing (NGS) platform (Thermo Fisher Scientific, Waltham, MA, USA), as described previously [7, 16–18]. In more detail, we analyzed the whole coding region of *BCOR, BCORL1, CEBPA, DDX41, DNMT3A, ELANE, ETNK1, ETV6, GATA2, GNB1, HAX1, NF1, PHF6, PIGA, PPM1D, PRPF8, SF3B2, SFRP1, SRP72, STAG2, TP53,* and *ZRSR2*, as well as mutational hotspot regions of *NPM1, ASXL1, BRAF, CALR, CBL, CSF3R, CXCR4, ETNK1, EZH2, FLT3, IDH1, IDH2, JAK 2, KIT, KRAS, MPL, NRAS, PTPN11, RUNX1, SETBP1, SF3B1, SRSF2, STAT3, STAT5B, TET2, U2AF1* and *WT1 *genes. All diagnostic, treatment and outcome data and the results from NGS were retrieved from openMEDOCS, a regional hospital-based documentation system and continuously recorded in a dedicated internal database (Research, Documentation and Analysis database, RDA; https://imi.medunigraz.at/services).

Importantly, we also aimed for continuous biobanking of high-quality diagnostic and follow-up leukemia samples. Therefore, leukemic blasts were isolated from peripheral blood and/or BM by Ficoll (GE HealthCare, Chicago, IL, USA) density gradient centrifugation and subsequently stored in DMSO in the vapor phase of liquid nitrogen (Supplementary Fig. 1) [19–21].

The study was approved by the institutional review board of the Med Uni Graz (EK 30-464 ex 17/18 and EK 35-079 ex 22/23). Every patient signed an informed consent for data recording and analysis as well as for the biobanking of the respective specimens.

For statistical analysis, group differences in continuous variables were compared by Mann-Whitney U test. In contrast, Fisher’s exact test was employed to compare all dichotomous variables in patient specimens. Kaplan-Meier survival curves were used to generate figures for overall survival; differences between groups were assessed using the log-rank test. Univariable Cox proportional hazards models were used to investigate the association of group (study cohort, NGS cohort) and other parameters with the incidence of all-cause mortality. A multivariable analysis was conducted on parameters with *p* < 0.20. Hazard ratio (HR) with 95% confidence interval (CI) are reported. A *P*-value < 0.05 was considered to be statistically significant. The analyses were performed with GraphPad Prism vs. 10.1.2 (Boston, MA, USA) while the statistical software R (version 4.3.2; https://www.R-project.org/) with the survival package (version 3.5‑7; https://CRAN.R-project.org/package=survival) was used for the survival analysis (Cox models).

## Results

### Description of the cohort

The study cohort consisted of 447 consecutive AML patients treated at the Clinical Division of Hematology, Med Uni Graz, Austria. In more detail, we included the data from 284 patients treated in the NGS era (2013–2023) and 163 patients treated before this time (2002–2008). Detailed clinical characteristics are depicted in Supplementary Tables 1 and 2.

### Application and results of NGS

**Fig. 1:**
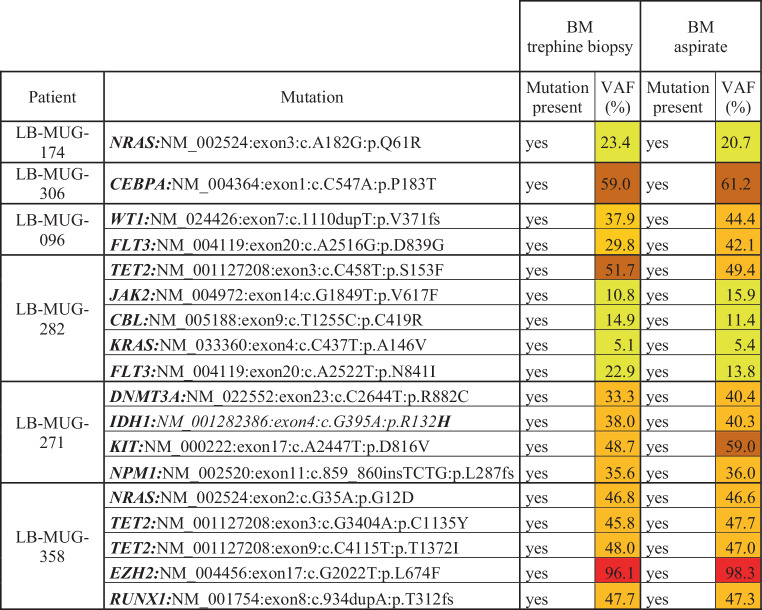
Comparison of NGS results from BM trephine biopsies and aspirates. For better visibility and comparability between BM trephine biopsies and aspirates, VAF < 25% are highlighted in *yellow*, VAF ≥ 25% and < 50% in *light orange*, VAF ≥ 50% and < 75% in *dark orange*, and VAF ≥ 75% *in red*. *NGS* next-generation sequencing, *LB-MUG* Leukemia Biobank Med Uni Graz, *BM* bone marrow, *VAF* variant allele frequency

A focus of this study was to evaluate the applicability and outcomes of NGS profiling within a routine clinical setting. The NGS has been available for the routine work-up of AML at the Institute of Pathology, Med Uni Graz since 2013. It was ordered by the treating hematologist in 267/284 (94%) of patients. The cases where no NGS was ordered were predominantly patients treated with hypomethylating agents (HMA) or best supportive care (BSC) only, but also three individuals treated by intensive chemotherapy (ICT) diagnosed before 2016. While we primarily aimed for NGS analyses from BM aspirates, this procedure was not possible in all patients at diagnosis, i.e., in case of dry tabs. Therefore, we performed comparative experiments, analyzed BM trephine biopsies and corresponding aspirates from six patients, and observed comparable results (Fig. 1).

Consequently, we routinely performed NGS from trephine biopsies in case of missing or insufficient aspirates. This led to successful NGS analyses in all patients allocated for sequencing at the Med Uni Graz between 2013–2023 included in this study. As a comparison, the combination of cytogenetic analyses and targeted Fluorescence In Situ Hybridization (FISH) markers was attempted in all patients but yielded an evaluable result less frequently (259/284 cases, 91%, *P* < 0.0001). The reasons behind this were either dry tap bone marrow aspirations or missing growth of cells that did not allow G‑banding. Of importance for the routine clinical setting, 10 patients with missing cytogenetics could still be risk-stratified, according to ELN2022, using molecular NGS techniques from the BM. This increased diagnostic accuracy is due to the detection of mutated *TP53* in sequencing and the identification of prognostically determining translocations in NGS-based RNA translocation analyses, as described by our group previously [7]. We then assessed the duration from BM sampling until the availability of NGS results. The average time for this period was 16 days, with decreasing values from 2013/2014 (22 days) to 2022 (10 days). Of note, the average time from BM sampling until the availability of cytogenetic results was comparable to NGS (18 days) with decreasing values from 2013/2014 (26 days) to 2022 (11 days). Another important aspect of NGS usefulness in the clinical routine is its potential to identify targetable molecular lesions. Currently, these include mutations in *FLT3, IDH1* and *IDH2*, where drugs licensed by the European Medicines Agency (EMA) and/or the U.S. Food and Drug Administration (FDA) are available. Altogether, within our cohort, NGS identified mutations within these genes in 107/284 (38%) of patients, further supporting its relevance for the routine clinical management of AML.

**Fig. 2:**
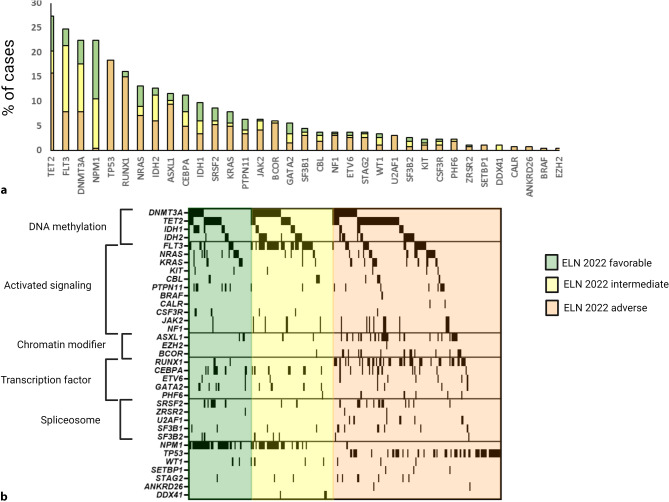
Molecular landscape of 267 AML patients analyzed with NGS.** a** Mutation frequencies. **b** Oncoplot showing nonsynonymous mutations in individual genes, grouped into categories, as labeled on the left. Every column on the x-axis represents a single patient. Colors reflect the ELN2022 risk groups. Only genes with at least one mutation are shown

Concerning the molecular profile observed, we corroborated previously published mutational landscapes of AML (Fig. 2). The most frequently mutated genes included *TET2* (*n* = 73/267, 27%), *FLT3* (*n* = 66/267, 25%), *DNMT3A* (*n* = 60/267, 23%, and *NPM1* (*n* = 60/267, 23%) [22–25]. Importantly, our study also includes NGS data from older patients treated with nonintensive chemotherapy (NICT) or BSC only. Therefore, our data reflect a real-world perspective of AML, not biased by the potential inclusion criteria of CCITs. When comparing older patients ≥ 70 years (median 77 years, range 70–88 years) with younger patients < 70 years (median 59 years, range 19–69 years), we found an enrichment of mutations in *IDH2, SRSF2, TET2, *and *TP53* in the older patient cohort (*IDH2*: 18/81, 22% in older patients vs. 16/186, 9% in younger patients *P* = 0.004; *SRSF2*: 15/81, 19% in older patients vs. 9/186, 5% in younger patients *P* < 0.001; *TET2*: 31/81, 38% in older patients vs. 42/186, 23% in younger patients *P* = 0.011; *TP53*: 21/81, 26% in older patients vs. 28/186, 15% in younger patients *P* = 0.040). With respect to mutation categories, we observed an increased frequency of mutations affecting DNA methylation and the spliceosome in the older patients (DNA methylation *DNMT3A, TET2, IDH1/2*: 58/81, 72% in older patients vs. 84/186, 45% in younger patients *P* < 0.001; spliceosome *SRSF2, ZRSR2, U2AF1, SF3B1, SF3B2*: 23/81, 28% in older patients vs. 21/186, 11% in younger patients *P* = 0.006). On the contrary, mutations affecting cellular signalling were more frequent in younger patients (*FLT3, NRAS, KRAS, KIT, CBL, PTPN11, BRAF, CALR, CSF3R, JAK 2, NF1*; 37/81, 46% in older patients vs. 114/186, 61% in younger patients *P* = 0.022). We also observed enrichment of MDS-related mutations according to the World Health Organization (WHO) 2022 classification [26] in older patients (33/81, 41% in older patients vs. 48/186, 26% in younger patients *P* = 0.020). The NGS distribution data and mutation heatmaps of older and younger patients are presented in Table 1 and Supplementary Figures 2–3.

**Table 1:** Mutation frequencies in older and younger AML patients

Gene/gene group	≥ 70 years	< 70 years	*P*-value
*ANKRD26*	0/81 (0%)	2/186 (1%)	> 0.999
*ASXL1*	12/81 (15%)	20/186 (11%)	0.412
*BCOR*	3/81 (4%)	14/186 (8%)	0.288
*BRAF*	1/81 (1%)	0/186 (0%)	0.303
*CALR*	0/81 (0%)	2/186 (1%)	> 0.999
*CBL*	1/81 (1%)	10/186 (5%)	0.181
*CEBPA*	12/81 (15%)	19/186 (10%)	0.302
*CSF3R*	2/81 (2%)	4/186 (2%)	> 0.999
*DDX41*	0/81 (0%)	3/186 (2%)	0.248
*DNMT3A*	18/81 (22%)	43/186 (23%)	> 0.999
*ETV6*	1/81 (1%)	9/186 (5%)	0.291
*EZH2*	1/81 (1%)	0/186 (0%)	0.303
*FLT3*	15/81 (19%)	51/186 (27%)	0.127
*GATA2*	3/81 (4%)	12/186 (6%)	0.564
*IDH1*	12/81 (15%)	16/186 (9%)	0.134
***IDH2***	**18/81 (22%)**	**16/186 (9%)**	0.004**
*JAK 2*	6/81 (7%)	11/186 (6%)	0.599
*KIT*	0/81 (0%)	6/186 (3%)	0.182
*KRAS*	3/81 (4%)	18/186 (10%)	0.137
*NF1*	3/81 (4%)	8/186 (4%)	> 0.999
*NPM1*	18/81 (22%)	42/186 (23%)	> 0.999
*NRAS*	9/81 (11%)	26/186 (14%)	0.694
*PHF6*	0/81 (0%)	6/186 (3%)	0.182
*PTPN11*	3/81 (4%)	14/186 (8%)	0.288
*RUNX1*	13/81 (16%)	31/186 (17%)	> 0.999
*SETBP1*	1/81 (1%)	2/186 (1%)	> 0.999
*SF3B1*	5/81 (6%)	7/186 (4%)	0.521
*SF3B2*	1/81 (1%)	7/186 (4%)	0.442
***SRSF2***	**15/81 (19%)**	**9/186 (5%)**	**<** 0.001**
*STAG2*	5/81 (6%)	5/186 (3%)	0.177
***TET2***	**31/81 (38%)**	**42/186 (23%)**	0.011*
***TP53***	**21/81 (26%)**	**28/186 (15%)**	0.040*
*U2AF1*	3/81 (4%)	5/186 (3%)	0.702
*WT1*	1/81 (1%)	8/186 (4%)	0.285
*ZRSR2*	2/81 (2%)	1/186 (1%)	0.219
**DNA methylation**	**58/81 (72%)**	**84/186 (45%)**	**<** 0.001**
**Activated signaling**	**37/81 (46%)**	**114/186 (61%)**	0.022*
Chromatin modifier	14/81 (17%)	31/186 (17%)	> 0.999
Transcription factor	23/81 (28%)	63/186 (34%)	0.397
**Spliceosome**	**23/81 (28%)**	**21/186 (11%)**	0.006**
**MDS-related WHO 2022**	**33/81 (41%)**	**48/186 (26%)**	0.020*
MDS-related ICC 2022	37/81 (46%)	65/186 (35%)	0.102

DNA methylation: *DNMT3A, TET2, IDH1/2; *activated signaling*: FLT3, NRAS, KRAS, KIT, CBL, PTPN11, BRAF, CALR, CSF3R, JAK 2, NF1; *chromatin modifier*: ASXL1, EZH2, BCOR; *transcription factor*: RUNX1, CEBPA, ETV6, GATA2, PHF6; *spliceosome*: SRSF2, ZRSR2, U2AF1, SF3B1, SF3B2*

Parameters with statistical significance were highlighted in bold. * = *P*-value < 0.05, ** = *P*-value < 0.01.

### Progress in AML treatment over time

**Fig. 3:**
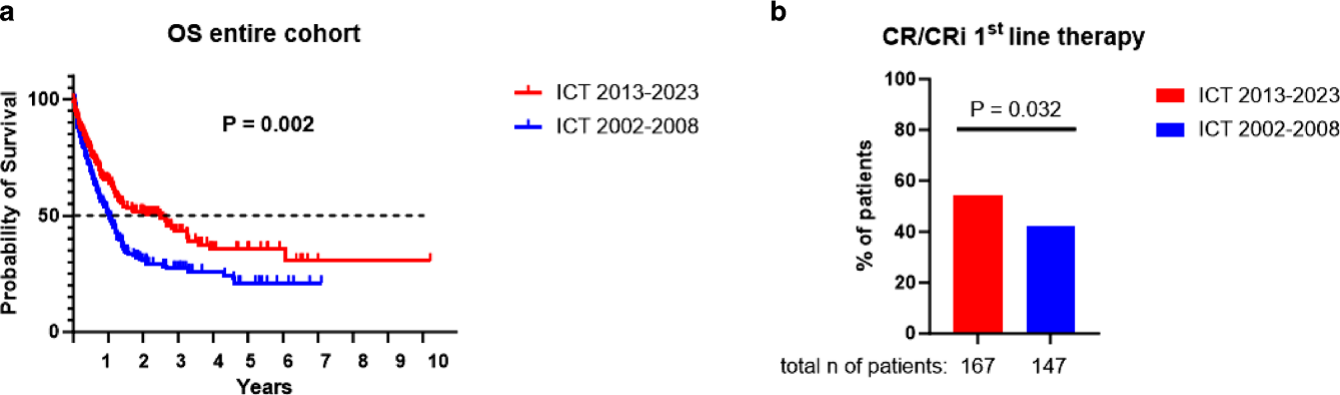
Comparison between the NGS-era (2013–2023) and the pre-NGS era (2002–2008).** a** Comparison of overall survival between patients treated with intensive chemotherapy (ICT). **b** Cumulative CR/CRi rates in patients treated with ICT between 2002–2008 and 2013–2023. Results of multivariable analyses are displayed in Supplementary Table 3

In the next step, we were interested in whether the outcome of AML patients improved in the NGS era. Therefore, we compared treatment outcomes to a cohort of 163 unselected, consecutive patients treated between 2002–2008 at our division. Of note, neither NGS nor molecularly targeted therapies were available at this time. Except for low-dose Ara‑C (LDAC), non-intensive AML treatment options were also not available. Therefore, for better comparison, we focused our analyses only on intensively treated patients receiving 7 + 3 based regimens ± allogeneic hematopoietic stem cell transplantation (allo-HSCT) and compared 167 patients treated between 2013–2023 with 163 cases treated between 2002–2008. We first compared overall survival (OS) and observed a significant elongation in patients treated between 2013–2023 (median survival 910 days for 2013–2023 vs. 371 days for 2002–2008; *P* = 0.002; Fig. 3a) with a hazard ratio (HR) of 0.65 (95% confidence interval, CI 0.49–0.86). When searching for potential reasons behind improved OS in the 2013–2023 cohort, descriptive statistics revealed an increased rate of allografting and a higher rate of complete remissions (CR)/CR with incomplete count recovery (CRi) to first line therapy within these patients, while all other factors assessed (age, sex, white blood cell count, WBC, peripheral blood, PB, blasts and lactate dehydrogenase, LDH) were similarly distributed between the groups (Supplementary Table 2). Importantly, allo-HSCT and CR/CRi to first line therapy were associated with prolonged survival in univariable analyses and remained independent significant factors in multivariable models (Supplementary Table 3). Hence, these data suggest that the better survival in the 2013–2023 cohort is mainly mediated through a higher transplantation frequency and a better treatment response to first line regimens. Interestingly, the median age at transplantation was also higher in the 2013–2023 cohort (2002–2008: median 46 years, range 18–68 years; 2013–2023: median 56 years, range 19–71 years; *P* < 0.001), reflecting the clinical experience that allo-HSCT is more often offered to older patients nowadays. We did not have sufficient information on the cytogenetic analyses in the 2002–2008 cohort, precluding comparison of specific karyotype-defined risk groups between the 2002–2008 and 2013–2023 cohorts.

### Progress in AML treatment

Our data reveal increased use of allo-HSCT and higher rates of CR/CRi after first line therapy as major determinants of improved survival in recent times. Considering higher response rates to first line therapy, it could be hypothesized that this is caused by the advent of novel and molecularly targeted drugs that can be applied after NGS-based molecular characterization. To elaborate on this topic, we performed exploratory analyses studying potential effects of selected novel substances in the 2013–2023 cohort.

**Fig. 4:**
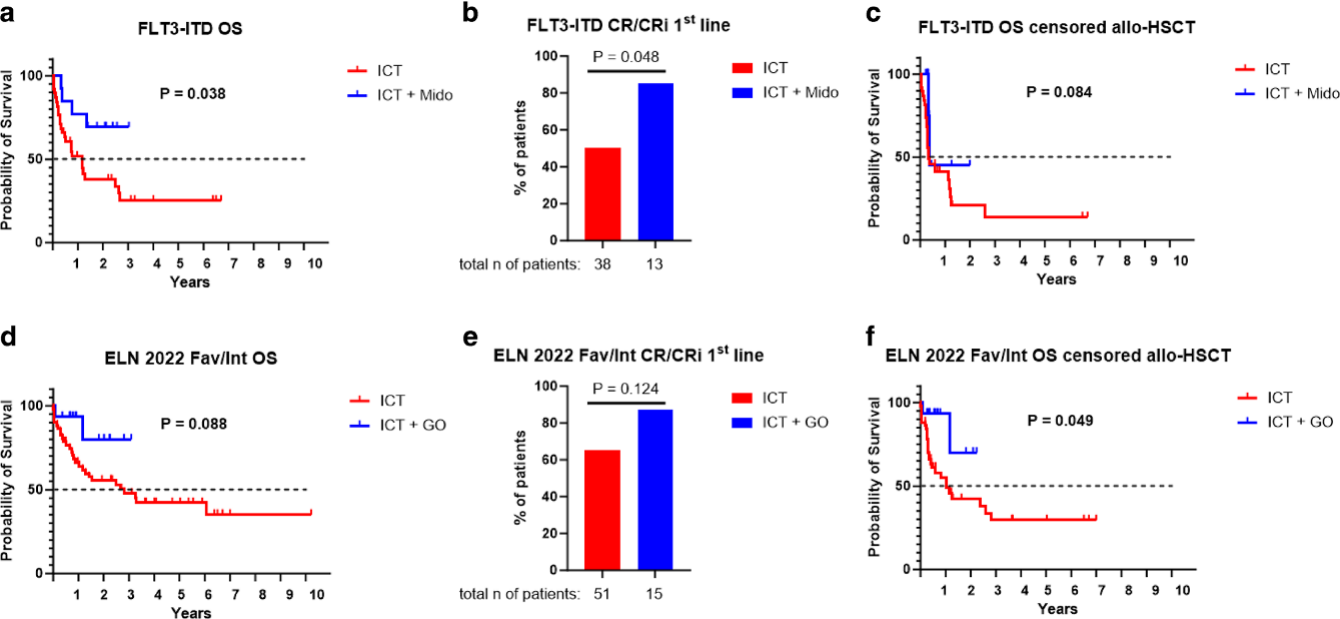
Addition of novel substances to ICT. Comparison of overall survival between patients treated with intensive chemotherapy (ICT) ± midostaurin (**a**, Mido) and ± gemutuzumab ozogamicin (**d,** GO). Comparison of overall survival censored for allogeneic hematopietic stem cell transplantation (allo-HSCT) between patients treated with ICT ± Mido (**b**) and ± GO (**e**). Cumulative CR/CRi rates in ICT-treated patients ± Mido (**c**) and ± GO (**f**)

Midostaurin is a FLT3 inhibitor and is nowadays added to 7 + 3-based ICT in *FLT3* mutated AML patients [8]. It was licensed by the EMA in 2017; therefore, the 2013–2023 cohort comprises *FLT3*-mutated patients treated with and without the addition of midostaurin (*n* = 13 patients ICT + midostaurin vs. 38 patients ICT only). When comparing the OS between these groups, patients treated with ICT and adding midostaurin had a significantly better survival (median survival 427 days for ICT vs. median survival not reached for ICT + midostaurin; *P* = 0.038; Fig. 4a). This improved outcome correlated with higher response rates to this therapy (first line CR/CRi rates 11/13, 85% for ICT + midostaurin vs. 19/38, 50% for ICT only; *P* = 0.048; Fig. 4b). Statistical significance got lost after censoring for allo-HSCT (median survival 126 days for ICT vs. 139 days for ICT + midostaurin; *P* = 0.0835; Fig. 4c); however, it has to be noted that the vast majority of *FLT3*-mutated patients were allografted in CR1 at our division.

Another targeted treatment approach licensed between 2013–2023 is gemtuzumab ozogamicin (GO). It is again added to ICT regimens and has been licensed for CD33-positive AML by the EMA in 2018 [27, 28]. The ALFA-0701 licensing trial showed the most prominent benefit of GO addition within the favorable and intermediate-risk groups [27]. Consequently, international guidelines like *Onkopedia* recommend its use only within these AML subgroups (https://www.onkopedia.com/). As this strategy is followed at our division, we focused on comparing intensively treated patients with and without GO in these risk groups (*n* = 15 patients ICT + GO vs. 51 patients ICT only). Addition of GO resulted in a trend to longer OS, although statistical significance was not reached (median survival 1025 days for ICT vs. median survival not reached for ICT + GO; *P* = 0.088; Fig. 4d). In agreement with the ALFA-0701 licensing trial [27], we failed to observe differences in CR/CRi rates between ICT and ICT + GO (first line CR/CRi rates 13/15, 87% for ICT + GO vs. 33/51, 65% for ICT only; *P* = 0.124, Fig. 4e); however, after censoring for allo-HSCT, the addition of GO to ICT correlated with a significant elongation of survival, suggesting that particularly patients without allografting profit from this combination (median survival 381 days for ICT vs. median survival not reached for ICT + GO; *P* = 0.049; Fig. 4f).

Other agents licensed between 2013–2023 were either not molecularly targeted (such as CPX-351 [29]), not available until the data cut-off in April 2023 (quizartinib) or not used as first line approaches in patients eligible for ICT and were therefore not analyzed in this study.

**Fig. 5:**
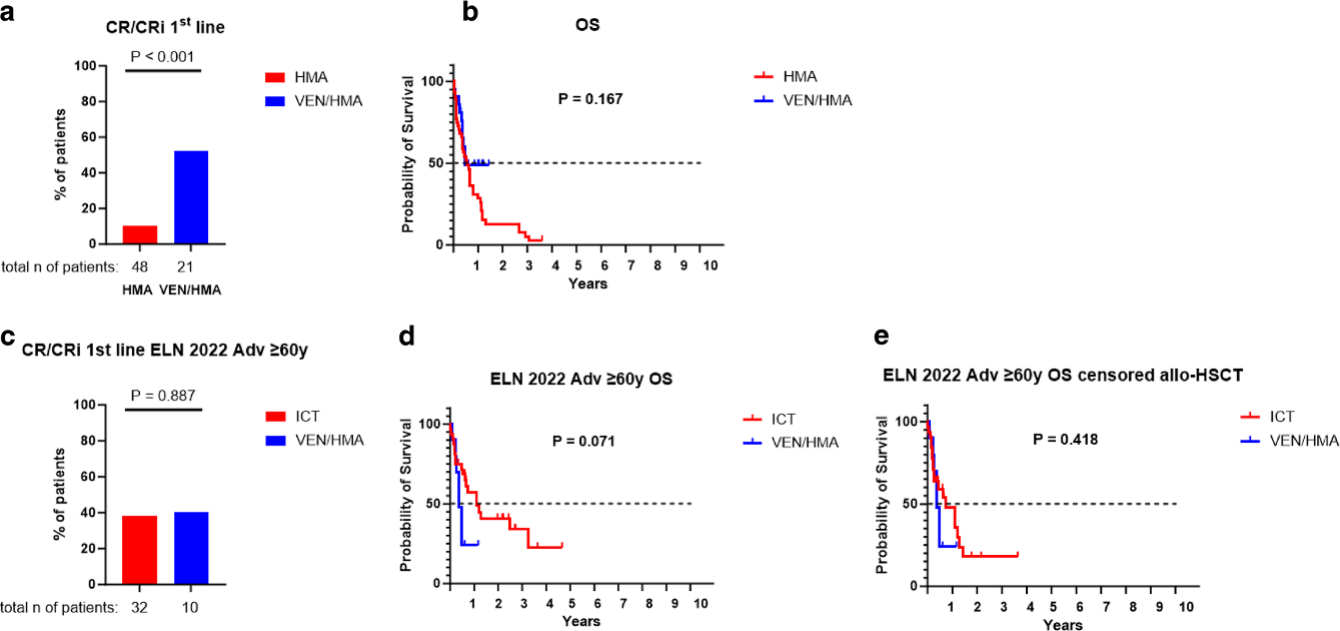
Outcome data of combining hypomethylating agents (HMA) with venetoclax (VEN). **a** Cumulative CR/CRi rates and **b** overall survival (OS) of patients treated with HMA monotherapy and HMA/VEN combination. **c** Cumulative CR/CRi rates in patients treated with ICT and VEN/HMA. **d** Comparison of OS between patients treated with intensive chemotherapy (ICT) and VEN/HMA. **e** Comparison of OS censored for allogeneic hematopoietic stem cell transplantation (allo-HSCT) between patients treated with ICT and VEN/HMA

For nonintensively treated patients, the hypomethylating agents azacitidine (AZA) and decitabine (DEC) were used throughout the 2013–2023 observation period [30–32]. Like all other treatments employed (ICT and venetoclax/HMA), HMA monotherapy also proved to be efficacious and prolonged the OS compared with best supportive care (Supplementary Fig. 4). In this respect, we did not observe a difference in CR/CRi rates or OS between the HMAs AZA and DEC (CR rates: 3/32, 9% for AZA vs. 2/16, 13% for DEC, *P* = 0.738; OS: median survival 198 days for AZA vs. 146 days for DEC, *P* = 0.872; Supplementary Fig. 5). Venetoclax (VEN) was added to eligible patients (see VIALE‑A licensing trial for details [14]) after its licensing by the EMA in 2021. We could compare the outcomes of 48 patients treated with HMA monotherapy and 21 patients treated with HMA/VEN. The CR/CRi rates were significantly improved in the HMA/VEN group (5/48, 10% for HMA vs. 11/21, 52% for HMA/VEN, *P* < 0.001; Fig. 5a). This improved treatment response did not correlate with a better OS in patients treated with HMA/VEN (median survival 198 days for HMA vs. 171 days for HMA/VEN, *P* = 0.167; Fig. 5b); however, this analysis might have been biased by the small cohort size and the short observational period in the HMA/VEN group. Due to missing EMA licensing, LDAC/VEN [33] was not used in our hospital. Moreover, other novel licensed and targeted treatment approaches (including ivosidenib for IDH1-mutated patients [13]) were not available during the observation period 2013–2023 (data cut-off 19 April 2023).

While HMA/VEN nowadays has been established as the gold standard for ICT-ineligible AML patients, recent data might also warrant its use in specific subgroups of patients eligible for ICT and allo-HSCT. Based on these data, the current National Comprehensive Cancer Network (NCCN) guidelines (version 4.2023, https://www.nccn.org/guidelines/category_1) enable its use in patients with poor risk constellation. This regimen has also been followed in our institution for older patients ≥ 60 years and adverse ELN2022 risk stratification and enabled the comparison of ICT and HMA/VEN within this high-risk cohort (*n* = 10 for HMA/VEN vs. *n* = 32 for ICT). Interestingly, CR/CRi rates were similar between the groups (12/32, 38% for ICT vs. 4/10, 40% for HMA/VEN, *P* = 0.887, Fig. 5c). Regarding OS, there was a trend for longer survival in patients treated with ICT; h, statistical significance was not reached (median survival 407 days for ICT vs. 136 days for HMA/VEN, *P* = 0.071, Fig. 5d). This trend to extended survival in patients treated with ICT was lost when OS was censored for allo-HSCT (median survival 275 days for ICT vs. 136 days for HMA/VEN, *P* = 0.418, Fig. 5e), suggesting that the improved survival of older adverse-risk patients treated with ICT might be mediated by consolidation with allo-HSCT.

## Discussion

In this study, we performed a retrospective and unselected data analysis of AML in patients treated within the NGS era at a tertiary care center. We aimed to record and analyze epidemiological data and treatment outcomes as part of a real-world observational study. In addition, we aimed to analyze the feasibility and results of NGS, particularly in patient groups not represented in CCIT. Moreover, we use this registry to analyze potential progress in AML treatment compared to data before the NGS era and/or before the introduction of recently licensed novel treatment approaches.

The use of NGS has revolutionized the diagnostics and risk stratification of AML and helped to identify novel targets for molecularly tailored treatment approaches. Consequently, it is a necessary tool for fulfilling the ELN 2022 risk stratification, where molecular information has gained importance compared to previous versions. Unfortunately, most NGS data describing the molecular landscape of AML are derived from patient cohorts treated within CCIT, and real-world data about its feasibility in the clinical routine and data about the molecular landscape of patients not included in CCIT are scarce. We performed a retrospective, unselected data analysis of 284 AML patients treated between 2013–2023 at a tertiary care cancer center. NGS was routinely performed at diagnosis during this time. We initially focused on the feasibility and benefits of NGS within a clinical routine setting. We primarily used BM aspirates for NGS analyses; however, in cases where aspirates were unavailable NGS was performed from formalin-fixed paraffin-embedded (FFPE) BM trephine biopsies. This strategy resulted in evaluable NGS results in all cases allocated to NGS analyses. Importantly, we also present data showing that NGS results from BM aspirates and FFPE trephine biopsies are comparable, indicating that our approach is feasible for a routine clinical setting. In this respect, it is also worth mentioning that NGS was successful significantly more often than conventional cytogenetics and that 10 cases with missing cytogenetics could still be risk-stratified according to the molecular analyses. Despite these results, it is important to emphasize that the joint assessment of NGS and cytogenetics is essential for accurate risk stratification in many patients, particularly as our data suggest that the period between BM sampling and availability of the results is comparable between these assays. Another aspect of NGS applicability in the clinical routine is the processing time, defined by the number of days passing between BM sampling and the availability of a finished report. We show that the median NGS processing time in a clinical routine setting is only 16 days and substantially decreased with the advances of NGS technology to 10 days in 2022. Considering recent observations that a delayed treatment start of more than 15 days in stable patients does not negatively affect outcomes [34], our data indicate that it is possible to wait for molecular profiling in a routine clinical setting. This statement is further supported by our observation that NGS identified a molecularly targetable lesion in 107/284 (38%) of patients. Taken together, our data clearly demonstrate that comprehensive NGS profiling is feasible within a routine clinical setting. It is a valuable tool for ELN risk stratification and targeted treatment decisions and should be an integral part of diagnosis at every center treating AML. In this respect, our data will provide argumentation help for implementation in those centers where organizational and/or financial issues precluded such an approach in the past.

Considering the molecular landscape of AML, we observed similar mutation patterns as described in CCIT and previous real-world cohorts [22–25]. This information is also relevant, as it strengthens the relevance and applicability of data from CCIT within this setting. Additionally, our molecular data confirm previous reports that the mutational landscape differs between younger and older patients. We have noted an increase of mutations in *TET2, IDH2, SRSRF2* and *TP53* in older patients, which matches well with previous reports [17, 25, 35–37]. The same is true when mutations were clustered to functional classes. In these analyses, we observed enrichment of mutations affecting DNA methylation and the spliceosome in old people, whereas aberrations activating cellular signalling were more frequent in younger patients, which matches well with the reported increased frequency of receptor tyrosine kinase and cellular signalling mutations in these age groups [25, 38]. Finally, we also observed higher frequencies of MDS-related mutations in old people, which is in accordance with data from clonal evolution data of AML [22, 26, 39, 40].

Also, regarding outcome data, our cohort matches well with internationally published data. Firstly, our analyses of OS again show that any treatment is better than no treatment and that survival can be extended with all treatment types currently available [41]. In this respect, we could also compare outcome data with a historical cohort of 163 AML patients treated at the same institution in the pre-NGS era. As low-intensity treatment with HMA or HMA/VEN was unavailable at this time, we focused on ICT regimens in these analyses. In agreement with other registries, we show that survival has significantly improved in recent times [5, 42, 43]. While there will certainly be an influence of improved supportive medicine, including ameliorated intensive care treatment plans for AML patients, our subanalyses demonstrated that the increased use of allografting and better response rates to first line therapy are major determinants of this success. Considering allo-HSCT, it might be postulated that improved risk stratification through NGS helped to better identify patients for allografting. In addition, we show that the increased frequency of allo-HSCTs performed in the more recent cohort is also due to a higher age limit for this complex therapeutic approach. Indeed, median age at transplantation was significantly higher in the more recently treated cohort. Considering improved response rates in more recent times, we have also seen the advent of novel molecularly targeted treatment approaches in the NGS era. We have performed exploratory analyses and tested two of these substances, the FLT3 inhibitor midostaurin and the CD33 antibody-drug conjugate GO; these drugs are added to conventional ICT regimens [8, 27, 28]. Midostaurin improved CR/CRi rates and survival, whereas GO did not affect CR/CRi rates but extended survival in favorable/intermediate risk patients without allo-HSCT consolidation. Hence, our data suggest that improved survival might not only mediated by the increased frequency of allografting but also by the success of novel and molecularly targeted treatment approaches. Of course, the smaller sample sizes of the tested cohorts precluding multivariable calculations limit these analyses and necessitate further validations in independent cohorts. One issue to discuss at this point is the observation that midostaurin improved CR/CRi rates in our study. In contrast, no effect on CR rates was seen in the phase 3 RATIFY licensing trial [8]. Interestingly, other real-world observations agree with our study and have seen the same contradictory results with higher CR/CRi rates in the patients treated with midostaurin [44]. As stated in this paper, a possible explanation for this disagreement with the RATIFY trial is that our trial used CR and CRi to define CR rates. In contrast, the RATIFY trial used CR only. Another potential reason might be due to the definition of CR. The RATIFY trial followed a stringent CR definition by only counting CRs occurring on or before 60 days of starting therapy (including two induction cycles); however, an expanded CR definition (CRs during protocol treatment and those in the 30 days following treatment discontinuation) was used in additional subanalyses. It showed significantly higher CR rates in patients randomized to midostaurin compared to placebo [8]. In our study, several CR/CRis occurred during this expanded CR definition, which might also contribute to this discrepancy. Finally, the limitation of our small sample size might have contributed to this discrepancy compared to the RATIFY trial results.

Our database also allowed us to analyze NICT regimens in older and unfit patients. As seen in the recently published VIALE‑A trial [14], adding the BCL‑2 inhibitor venetoclax to HMAs significantly improved the outcome compared to HMAs alone. The success of HMA/VEN is also relevant as it is increasingly used in fit patients replacing ICT. The recent NCCN guidelines for AML enable the use of HMA/VEN in patients with adverse risk stratification [45] as the outcome with ICT is unsatisfactory in these patient cohorts. A series of retrospective real-world data support this approach [46–49]. Our registry also enabled us to address this issue by comparing the differences between HMA/VEN and ICT in older patients ≥ 60 years and ELN2022 adverse risk. When analyzing OS, we observed that patients treated with ICT performed significantly better; however, when data were censored for allo-HSCT, the advantage of ICT was lost, suggesting that the extended OS in patients treated with ICT within this cohort is mainly mediated through allografting. In agreement with these data, CR/CRi rates were comparable between these groups. Although it has to be noted that these data are limited by the small number of patients treated with HMA/VEN, they further support the NCCN guidelines and the abovementioned retrospective observations. They might lead to the hypothesis that a paradigm shift for older patients with adverse risk classification from ICT-based regimens to the NICT HMA/VEN could happen soon. This could also hold for patients still eligible for allo-HSCT, with HMA/VEN used as an induction regimen instead of ICT. Whether HMA/VEN should be used as maintenance after allografting in such a scenario cannot be answered with this registry and is currently being addressed within the VIALE‑T trial. Of note, HMA/VEN was not used in ICT-eligible patients with ELN2022 intermediate and favorable risk; therefore, conclusions about a comparison between ICT and HMA/VEN outside the adverse risk group cannot be drawn from this study.

Ultimately, we could connect this registry to a state of the art leukemia biobank, which enables the corroboration of therapy-relevant molecular findings, familial analyses, and the establishment of novel methods. In addition, it is a precious resource for leukemia research and is open for internal and external collaboration projects.

Taken together, we present a comprehensive real-world registry of AML patients treated at a tertiary care cancer center in the NGS era that is additionally linked to a leukemia biobank containing high-quality biospecimens of these patients. The registry is a crucial tool for quality control and assurance and also helps to validate data from CCIT in a real-world setting. These data might even help to approach clinical questions that have not been studied in CCIT yet and design relevant CCITs for these questions. One example is the treatment of older patients with adverse risk stratification with ICT or HMA/VEN, where our data support current NCCN guidelines [45] and other real-world registries [46–49] and suggest HMA/VEN as a valuable option in these patients. Finally, our data report important confirmatory data considering the molecular landscape in AML patients and underline the importance of routinely performed NGS profiling at diagnosis for correct diagnosis, risk stratification, and treatment planning. Despite all these benefits, mentioning this study’s limitations is important. Firstly, the retrospective nature of this study introduces a potential bias regarding patient selection, unequal treatment regimens, maintenance treatment, and others. Additionally, the limited number of patients in this study, particularly in some subgroups, which also precluded multivariable models assessing the impact of confounding factors, is another limiting factor. This is particularly true for analyses of novel agents, including midostaurin, GO, and HMA/VEN (the latter particularly in the adverse risk group). Furthermore, the follow-up of these cohorts is shorter than in the ICT group, introducing another potential bias.

## Supplementary Information

Supplementary tables with clinical data and statistical analyses and supplementary figures with molecular and outcome data

## Data Availability

The data that support the findings of this study are available on request from the corresponding author.
