# Supplementary material for: Acute myeloid leukemia in the next-generation sequencing era: Real-world data from an Austrian tertiary cancer care center
Source: Wien Klin Wochenschr. 2024 Nov 11;137(15-16):504–16. doi: 10.1007/s00508-024-02463-w (PMC12370787; doi:10.1007/s00508-024-02463-w)
Supplement: Supplementary file 1 — Supplementary tables with clinical data and statistical analyses and supplementary figures with molecular and outcome data [file 508_2024_2463_MOESM1_ESM.pdf]

**Supplementary material**

# Acute myeloid leukemia in the next-generation sequencing era: real-world data from an Austrian tertiary cancer care center

**Supplementary Tables**

| | **2013-2023 (n=284)** | **2002-2008 (n=163)** |
|---|---|---|
| **Age, years** | 65 (19-88) | 58 (18-79) |
| **Sex** | | |
| Male, n= | 156 (55%) | 83 (51%) |
| Female, n= | 128 (45%) | 80 (49%) |
| **WBC Count, x10$^9$/L** | 5.82 (0.47-388) | 10.95 (0.44-481) |
| **Hemoglobin, g/dL** | 9.2 (2.85-15.2) | n.a. |
| **Platelet Count, x10$^9$/L** | 56 (5-743) | n.a. |
| **Blasts (peripheral blood, %)** | 20 (0-98) | 12 (0-97) |
| **Blasts (bone marrow, %)** | 70 (0-100) | n.a. |
| **LDH U/L** | 354 (99-6213) | 386 (95-3696) |
| **ELN2022** | | |
| Favorable, n= | 53/269 (20%) | n.a. |
| Intermediate, n= | 67/269 (25%) | n.a. |
| Adverse, n= | 149/269 (55%) | n.a. |
| **ELN 2017** | | |
| Favorable, n= | 63/272 (23%) | n.a. |
| Intermediate, n= | 69/272 (25%) | n.a. |
| Adverse, n= | 140/272 (52%) | n.a. |
| **Therapeutic regimen** | | |
| ICT | 167/284 (59%) | 163/163 (100%) |
| VEN/HMA | 21/284 (7%) | n.a. |
| NICT | 60/284 (21%) | n.a. |
| BSC | 36/284 (13%) | n.a. |
| **allo-HSCT** | | |
| yes, n= | 100/284 (35%)<br>97/167 (58%) in ICT | 60/163 (37%) |
| no, n= | 184/284 (65%)<br>70/167 (42%) in ICT | 103/163 (63%) |

***Supplementary Table 1: Characterization of the 447 AML patients studied.*** *Continuous variables are displayed as median with the range. WBC, white blood cell count; LDH, lactate dehydrogenase; ELN, European LeukemiaNet; ICT, intensive chemotherapy; VEN, venetoclax; HMA, hypomethylating*

*agents; NICT, non-intensive chemotherapy; BSC, best supportive care; allo-HSCT, allogeneic stem cell transplantation; n.a., not available.*

| | **2013-2023 (n=167)** | **2002-2008 (n=163)** | **P-value** |
|---|---|---|---|
| **Age, years** | 58 (19-77) | 58 (18-79) | 0.7 |
| **Sex** | | | 0.3 |
| Male, n= | 95 (57%) | 83 (51%) | |
| Female, n= | 72 (43%) | 80 (49%) | |
| **WBC Count, x10$^9$/L** | 6.11 (0.47-388) | 10.95 (0.44-481) | 0.2 |
| **PB Blasts (%)** | 21 (0-98) | 12 (0-97) | 0.094 |
| **LDH U/L** | 352 (99-6213) | 386 (95-3696) | 0.7 |
| **CR/CRi 1$^{st}$ line** | | | 0.032 |
| Yes, n= | 91 (54%) | 62 (42%) | |
| No, n= | 76 (46%) | 85 (58%) | |
| Missing, n= | 0 | 16 | |
| **allo-HSCT** | | | <0.001 |
| Yes, n= | 97 (58%) | 60 (37%) | |
| No, n= | 70 (42%) | 103 (63%) | |

***Supplementary Table 2: Descriptive statistics of the 2002-2008 and 2013-2023 cohorts (only ICT-treated patients).*** *Continuous variables are displayed as median with the range. WBC, white blood cell count; PB, peripheral blood; LDH, lactate dehydrogenase; CR, complete remission; CRi, complete remission with incomplete count recovery; allo-HSCT, allogeneic stem cell transplantation.*

| | HR | 95% CI | P-value |
|---|---|---|---|
| **Cohort (2013-2023)** | 0.963 | 0.702-1.320 | 0.8 |
| **Sex (f)** | 0.771 | 0.569-1.045 | 0.094 |
| **CR/CRi 1st line (yes)** | 0.644 | 0.471-0.879 | 0.006 |
| **allo-HSCT (yes)** | 0.437 | 0.308-0.619 | <0.001 |
| **Age** | 1.015 | 1.002-1.028 | 0.023 |
| **WBC** | 1.001 | 0.998-1.005 | 0.5 |
| **LDH** | 1.000 | 1.000-1.000 | 0.016 |

***Supplementary Table 3: Multivariable analysis for overall survival.*** *All parameters with p-values <0.2 in univariable Cox proportional hazard analysis were included in this multivariable analysis. HR, Hazard ratio; CI, Confidence interval; f, female; WBC, white blood cell count; LDH, lactate dehydrogenase; CR, complete remission; CRi, complete remission with incomplete count recovery; allo-HSCT, allogeneic stem cell transplantation.*

**Supplementary Figures**

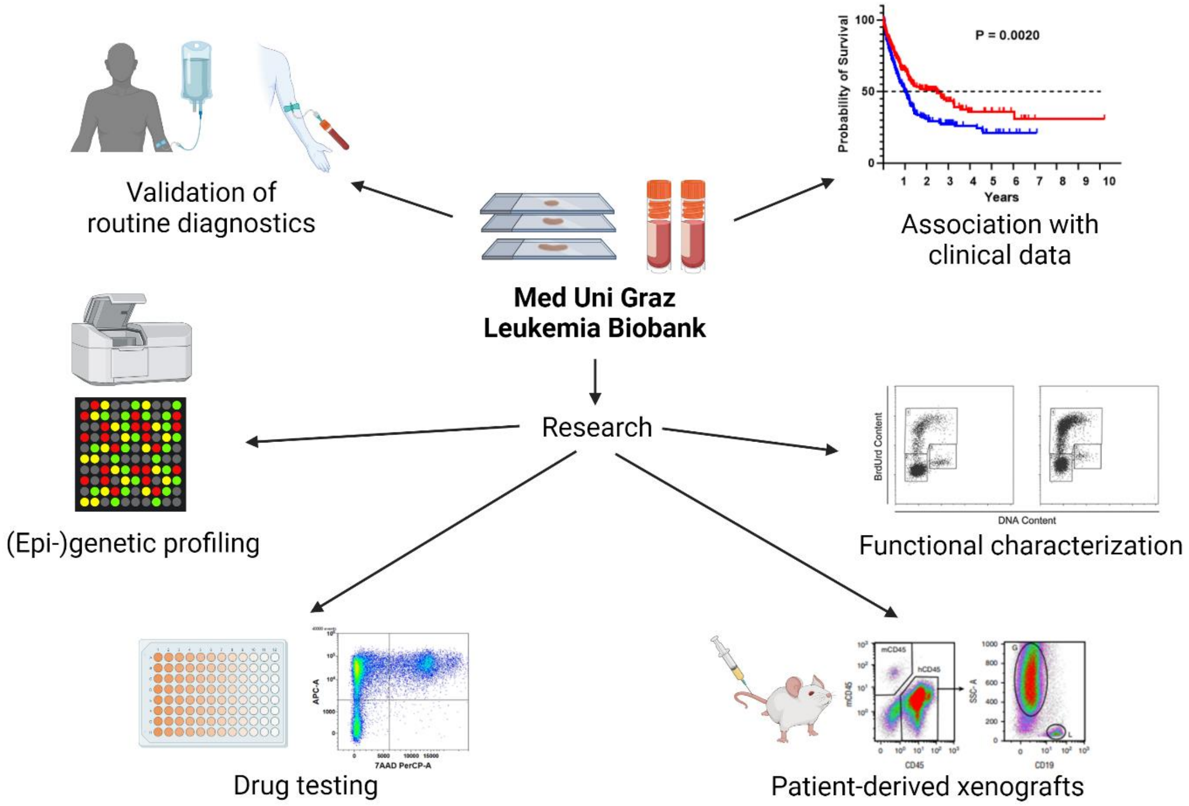


***Supplementary Figure 1: Med Uni Graz Leukemia Biobank.*** *Primary AML patient specimens at the Medical University of Graz are collected within the routine clinical setting and stored within a dedicated leukemia biobank. While these samples can be used for the validation and retrospective workup of specific clinical questions, they are also used for a series of scientific purposes. Importantly, the biobank is constantly linked to comprehensive clinical characterization of these patients. Finally, it is open for scientists at the Med Uni Graz and for national and international collaboration projects.*

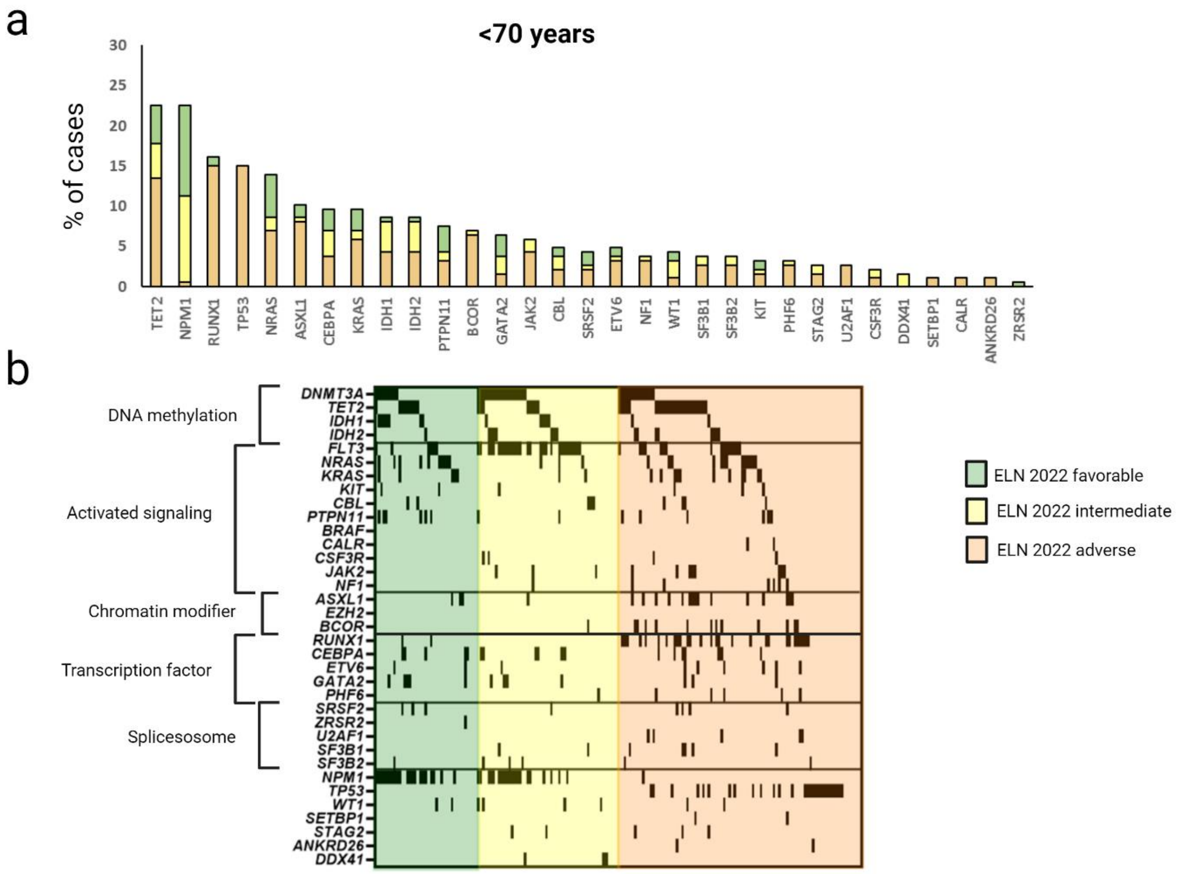


***Supplementary Figure 2: Molecular landscape of AML patients <70 years old (n=186). (a)*** *Mutation frequencies in younger patients <70 years of age.* ***(b)*** *Oncoplot showing nonsynonymous mutations in individual genes, grouped into categories, as labeled on the left. Every column on the x-axis represents a single patient. Colors reflect the ELN2022 risk groups. Only genes with at least one mutation are shown.*

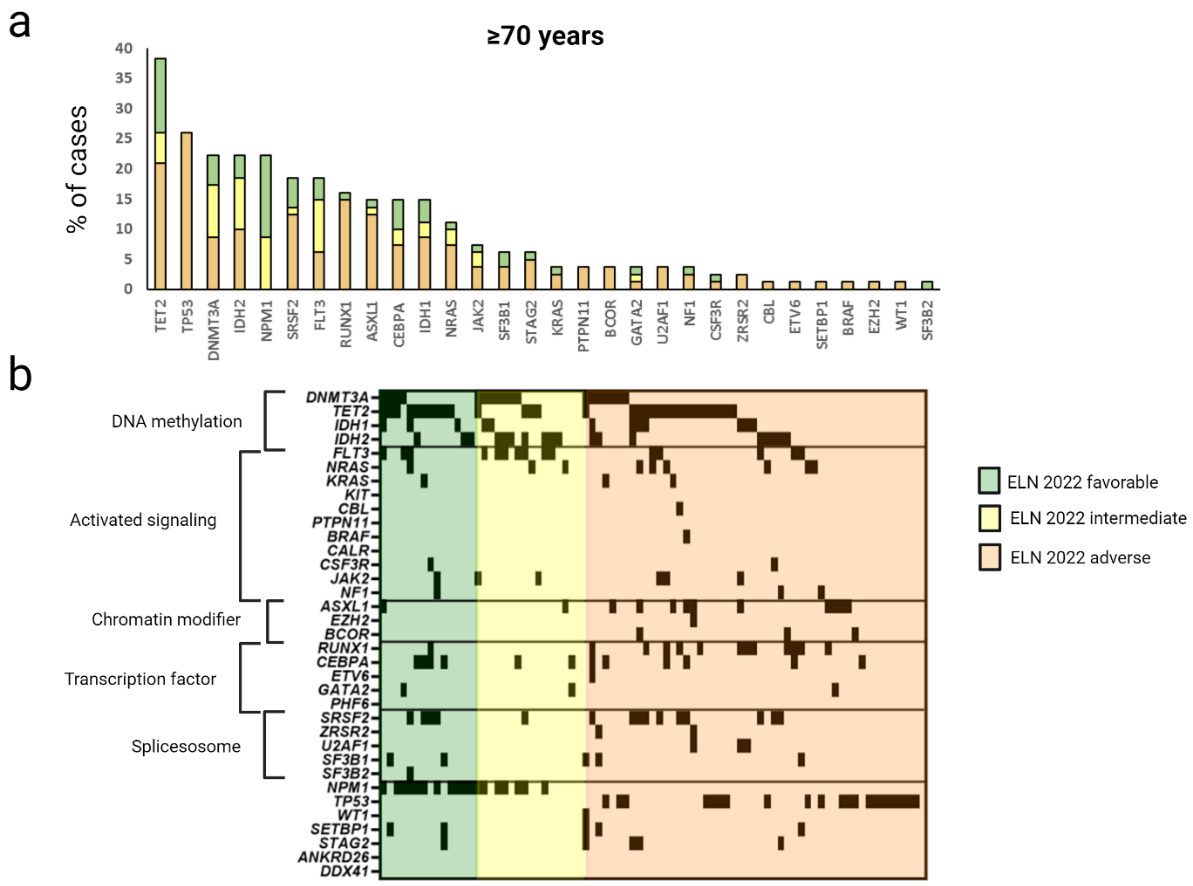


***Supplementary Figure 3: Molecular landscape of AML patients ≥70 years old (n=81). (a)*** *Mutation frequencies in older patients ≥70 years of age.* ***(b)*** *Oncoplot showing nonsynonymous mutations in individual genes, grouped into categories, as labeled on the left. Every column on the x-axis represents a single patient. Colors reflect the ELN2022 risk groups. Only genes with at least one mutation are shown.*

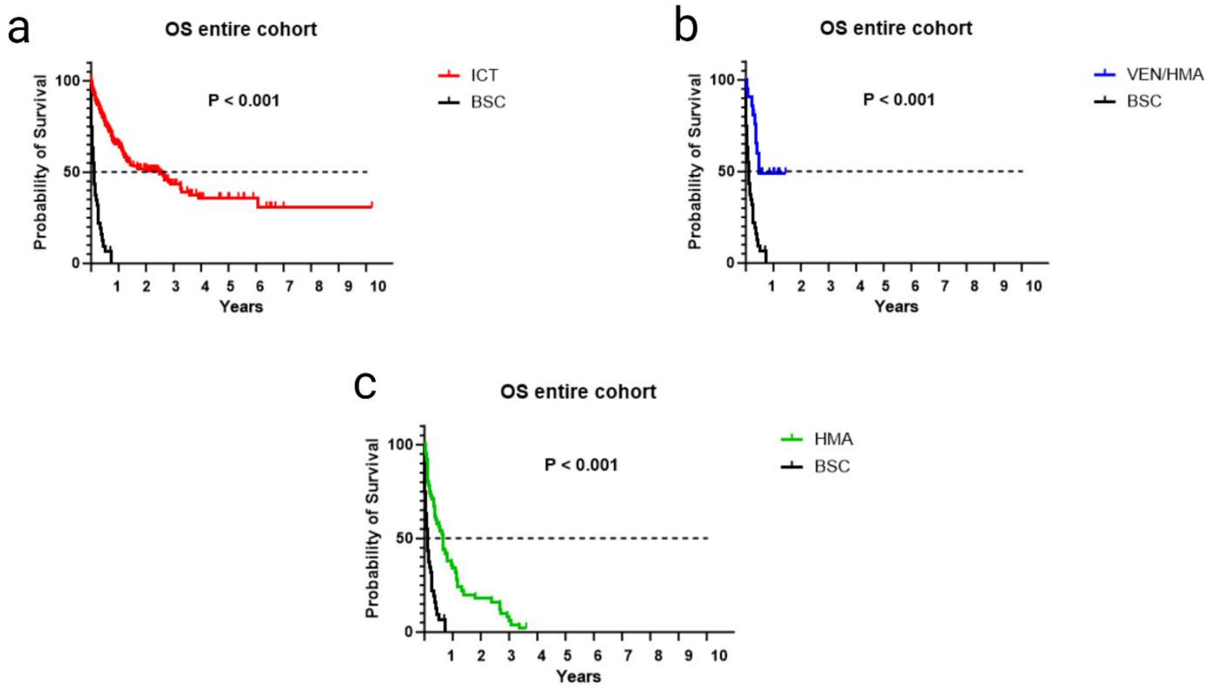


***Supplementary Figure 4: Comparison between therapy and best supportive care (BSC).*** *OS comparison between BSC and intensive chemotherapy (ICT, (**a**), Venetoclax/hypomathylating agents (VEN/HMA, **b**), and HMA (**c**).*

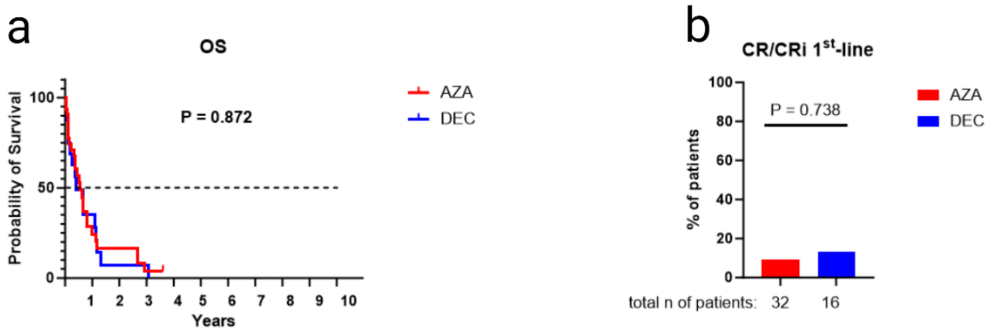


***Supplementary Figure 5: Comparison between the hypomethylating agents Azacitidine and Decitabine. a,*** *Comparison of overall survival between patients treated with Azacitdine (AZA) and Decitabine (DEC). **b,** Cumulative CR/CRi rates in AZA and DEC treated patients.*
